# Association of Periaortic Fat and Abdominal Visceral Fat with Coronary Artery Atherosclerosis in Chinese Middle Aged and Elderly Patients Undergoing Computed Tomography Coronary Angiography

**DOI:** 10.5334/gh.1078

**Published:** 2021-10-19

**Authors:** Jingqi Zhu, Zhangwei Yang, Xiaolin Li, Xiaoli Chen, Jingjiang Pi, Tao Zhuang, Jie Liu, Gang Li, Sheng Peng, Lin Zhang, Qi Zhang, Paul Chan, Brian Tomlinson, Huimin Fan, Liang Zheng, Zhongmin Liu, Yuzhen Zhang

**Affiliations:** 1 Department of Radiology, Shanghai Tenth People’s Hospital, Tongji University School of Medicine, Shanghai, CN; 2 Department of Radiology, Shanghai East Hospital, Tongji University School of Medicine, Shanghai, CN; 3 Jinggangshan University, school of medicine, Jiangxi, CN; 4 Key Laboratory of Arrhythmias of the Ministry of Education of China, Research Center for Translational Medicine, Shanghai East Hospital, Tongji University School of Medicine, Shanghai, CN; 5 Department of Cardiology, Shanghai East Hospital, Tongji University School of Medicine, Shanghai, CN; 6 Division of Cardiology, Department of Internal Medicine, Wan Fang Hospital, Taipei Medical University, Taipei, TW; 7 Faculty of Medicine, Macau University of Science and Technology, Macau, CN

**Keywords:** body ectopic fat distribution, periaortic fat, visceral fat, atherosclerosis, coronary artery disease

## Abstract

**Background and Aims:**

Coronary artery disease (CAD) is usually caused by atherosclerosis, which is associated with general obesity and stronger associations with localized ectopic fat depots have been reported. We measured body ectopic fat distribution in Chinese patients to determine the association with coronary artery atherosclerosis (CA).

**Methods:**

Patients undergoing coronary computed tomography angiography (CCTA) who agreed to participate in the study (n = 750, 50.4% men, mean age 64.8 years) had cardiovascular disease and risk assessment. Body ectopic fat depots were measured from CT and their association with CA, determined from CCTA, was evaluated by univariate and multivariate logistic regression models.

**Results:**

CAD with CA (CAD-CA) was present in 57.2% of participants with CAD of moderate/severe CA (CAD-msCA) present in 23.5% and both were significantly more frequent in men than in women. Overall, men had greater body mass index (BMI) but there was no difference in waist circumference (WC) between genders. However, significantly higher visceral adipose tissue (VAT) and periaortic fat volume (PAFV) were observed in men, whereas women had significantly higher abdominal subcutaneous adipose tissue (SAT). With increasing age, there was a significant decline in BMI, WC and SAT in men, but a significant increase of WC and VAT, PAFV and epicardial fat volume (EFV) in women. A high proportion of non-calcified plaques was observed in CAD-CA, 55.3% in CAD of minimal/mild CA (CAD-mmCA) with 38.7% exclusively non-calcified plaques, and 59.7% in CAD-msCA with multiple type plaques containing non-calcified ones. Multivariate logistic regression showed a significant association of PAFV with CAD-CA and CAD-msCA that was independent of general obesity and clinical risk factors, and independent of abdominal obesity in the highest PAFV quartile patients. VATA was associated with an increased prevalence of CAD-msCA in the patients in the upper 2 VATA quartiles that was independent of clinical risk factors and both general and abdominal obesity.

**Conclusions:**

We found age and gender differences of body ectopic fat distribution in Chinese patients with higher VAT and PAFV in men and higher SAT in women. With increased age, there was a decline of WC and SAT in men but not in women and an increase in WC, VAT and PAFV in women but not in men. PAFV was significantly associated with overall CAD-CA and CAD-msCA, while VAT was associated with CAD-msCA.

## Introduction

The prevalence of obesity is high and continues to increase worldwide [1]. According to the China Health and Nutrition Survey (CHNS) from 1989 to 2011, the age-adjusted prevalence of overweight, general obesity, and abdominal obesity in 2011 was 38.80%, 13.99% and 43.15% respectively, and significantly increased across all cycles of the survey among all subgroups [2]. Thus, the chronic diseases related to overweight and obesity have become a major health problem in China and globally.

Although body mass index (BMI) is a convenient way to monitor obesity prevalence at the population level, studies have shown that obesity defined by BMI is remarkably heterogeneous and that people with similar body weight or BMI values can have substantially different comorbidities and diverse levels of health risk [3,4]. Epidemiological evidences showed that the localized ectopic fat depots were associated with cardiovascular morbidity and mortality more strongly than increased total fat mass itself [5,6,7,8,9].

Abdominal obesity is prevalent in Chinese people and visceral adipose tissue (VAT) has been reported to have a stronger association with cardiovascular risk and cardiovascular diseases (CVD) than BMI [2,6,7], and it is a better predictor of incident CVD after adjustment for clinical risk factors and generalized adiposity [8,9]. More studies showed that the smaller ectopic fat depots including pericardial and periaortic fat were associated with cardiovascular risk factors and events [6,10], and pericardial fat, but not intrathoracic fat, was associated with coronary artery calcification, whereas intrathoracic fat, but not pericardial fat, was associated with abdominal aortic calcification after multivariable and VAT adjustment [10]. Furthermore, a multi-ethnic study of Caucasians, Filipinas and African-Americans supported that intra-thoracic fat was associated with the presence and severity of coronary artery calcium [11].

A multi-ethnic study of Caucasians, South Asians and African Caribbeans reported ethnic differences of fat deposition with South Asians having more and African Caribbeans less estimated VAT than Europeans [12], and both ethnic minorities having larger truncal skinfolds than Europeans. These differences in overall truncal fat, as well as VAT, contributed to the excess of diabetes in South Asian and African Caribbean groups, particularly for women. More recently, a Multiethnic Cohort Study of five different ethnic-racial groups showed extensive differences among ethnic-racial groups in the propensity to store fat intra-abdominally [13]. The visceral and liver fat were highest in Japanese Americans, lowest in African Americans, and intermediate in the other groups, and visceral and liver fat jointly accounted for a statistically significant fraction of the difference in the metabolic syndrome prevalence.

Despite the interest in body ectopic fat deposition contributing to obesity-related cardiometabolic risks and CVD in different ethnic groups, few studies have examined the ectopic fat depots which correlated with cardiovascular risks and CVD in Chinese populations [14,15,16]. China has experienced a fast economic development over the past three decades, while Shanghai has been one of the fastest developing cities in the world for the last twenty years resulting in a tremendous growth of CVD risk factors including overweight/obesity and their effects on cardiovascular health [17]. In the current study, we recruited middle aged and elderly outpatients, who visited Shanghai East Hospital and were referred for coronary computed tomography angiography (CCTA), for chest pain, check-up before surgery or routine check-up, to assess the body ectopic fat distribution with a non-contrast computed tomography (NCT) scan of the chest and abdomen, and coronary artery atherosclerosis (CA) with CCTA, and further evaluate the association of body ectopic fat deposition with CAD.

## Methods

### Data collection

The study was conducted in accordance with the Declaration of Helsinki and the Institutional Review Board of Shanghai East Hospital affiliated to Tongji Medical School approved the study protocol with serial number 2013-011. The written informed consent was obtained from each participant before any sample or data collection.

The inclusion criteria of the study population was all outpatients over 50 years old who visited Shanghai East Hospital during March 2013 to December 2014 for CCTA, and the exclusion criteria included the patients with heart failure, severe renal and hepatic dysfunction, symptomatic hyperthyroidism and Iodine allergy due to CCTA. The participants were instructed to come to the Department of Radiology in the morning after at least 10 hours of overnight fast for blood samples [18]. After blood sampling, the participants were interviewed by trained doctors for information on demographic characteristics, personal and medical history, and this was followed by anthropometric measurements, CT scan of the chest and abdomen and CCTA scan, and the measurement of body ectopic fat deposition and coronary artery atherosclerotic plaques was performed by trained specialists of Shanghai East Hospital.

### Imaging acquisition

#### Non-contrast-enhanced CT scans of the chest and abdomen for assessment of body ectopic fat depots and coronary artery calcium scores

All participants underwent CT scan of the chest and abdomen for assessment of body ectopic fat depots in the supine position using a dual-source CT scanner (Somatom Definition Flash, Siemens Healthcare, Forchheim, Germany). Only the participants with good image quality were included for further image evaluation.

For the thoracic image evaluation, all scans were performed during a single breath hold in an electrocardiogram (ECG)-triggering system with the acquisition window at 30%–70% of the R-R interval. The scan range extended from the level of the carina to 10 mm below the diaphragm for the general patients. However, for patients after coronary artery bypass graft (CABG), the scan range extended from 10 mm above the aortic arch to 10 mm below the diaphragm. For the abdominal image evaluation, all scans were performed during a single breath hold. The scan range extended from 10 mm above the diaphragm to the upper part of the iliac crest.

#### Coronary CT angiography (CCTA) for assessment of coronary artery atherosclerotic plaques

CCTA scan was performed with an ECG-gating system to enable retrospective registration of image reconstruction in the appropriate cardiac phase (at 30%–70% of the R-R interval) during a single breath hold. For the contrast-enhanced scan, 70–90 ml of a nonionic contrast agent (Omnipaque, 350 mgI/ml; GE Healthcare, Princeton, NJ, USA) was injected into an antecubital vein at a flow rate of 4–5 ml/s using a power injector, followed by 30 ml of saline. A bolus tracking technique was used to determine the delay time. The actual scan was started when the region of interest (ROI) within the ascending aorta reached a threshold of 120 Hounsfield units (HU). A medium-smooth convolution kernel (B26f) was chosen for the image reconstruction.

### Imaging analysis

#### Body fat measurements

The body ectopic fat depots were analyzed separately by two experienced readers (J.Z., Z.Y.) and the average values were used for analysis. For the inter-observer agreement of the measurements, we randomly sampled 30 patients among the participants and the quantifications were performed by J.Z., Z.Y. to determine each intraclass correlation (ICC) of the quantifications. The results were shown in Supplement Table 1 indicating consistent measurement by two doctors, and the sample figures were shown in Supplement Figure 1.

Visceral adipose tissue area (VATA) was defined as adipose tissue around the abdominal viscera and subcutaneous adipose tissue area (SATA) as adipose tissue outside the abdominal muscular wall at the level of the umbilicus. Visceral adipose tissue volume (VATV) and subcutaneous adipose tissue volume (SATV) were defined as adipose tissue around the abdominal viscera and outside the abdominal muscular wall between the level of diaphragm and low level of L3 vertebra, respectively. Waist circumference (WC) was measured by starting at the top of the hip bone, then bringing the tape all the way around the body level with the umbilicus.

Periaortic fat volume (PAFV) was defined as the adipose tissue around the thoracic aorta with the anatomic borders: 1) superiorly, the right pulmonary artery; 2) inferiorly, the diaphragm; 3) anteriorly, the esophagus; 4) posteriorly, bilateral costo-vertebral joints. Epicardial fat volume (EFV) was defined as the visceral intrapericardial fat contiguous with the myocardial surface, and paracardial fat volume (PaCFV) as the fat deposits in the mediastinum outside the parietal pericardium. PaCFV and EFV were measured by calculating the sum of the corresponding volumes from the right pulmonary artery to the diaphragm.

All of the body fat, as measured by CT, was defined as any pixel with a density between –195 and –45 HU within the ROI [9]. A semiautomatic technique for fat quantification was performed on the CT images with the use of software (Volume, Siemens Healthcare).

The mean liver attenuation was obtained from an average of three selected areas of approximately 200 mm^2^ at each ROI, including the right-anterior lobe, right-posterior lobe, and left lobe of the liver, at the level of the 12^th^ thoracic vertebra (T12). Also, the mean spleen attenuation was obtained from an average of two selected areas of approximately 200 mm^2^ at each ROI, including the anterior and posterior parts of the spleen at the same level as the liver. Finally, the liver-to-spleen attenuation ratio (LSR) was calculated and the liver fat was defined as liver attenuation ≤ 40 HU or LSR ≤ 1.10 [19].

### CCTA analysis

Coronary artery calcium (CAC) scores and CCTA datasets were analyzed separately by two experienced readers (J.Z., Z.Y.) in consensus using the same 15-segment American Heart Association model and the average values for further analysis with each ICC of the quantifications shown in Supplement Table 1 indicating consistent measurements [20]. CAC scores were quantified by the calcifications in the coronary arteries of NCT images with a detection threshold of 130 HU. Coronary artery atherosclerotic plaques were quantified with the aid of cardiac software (Circulation, Siemens Healthcare) and classified as follows: 1) calcified plaque (≥130 HU); 2) non-calcified plaque (<130 HU); or 3) mixed plaque (inclusive of both non calcified and calcified plaque components) [21].

### Study-outcome definitions

Body mass index (BMI) was defined as weight in kilograms divided by height in meters squared. Participants were classified as being generally overweight (BMI ≥ 24 and < 28 kg/m^2^) or obese (BMI ≥ 28 kg/m^2^) according to the Asian-specific BMI cut-points of the Health Standard of the People’s Republic of China National Health and Family Planning Commission [22]. Definite hypertension was defined as an average of two measurements of systolic blood pressure (SBP) ≥ 140 mmHg or diastolic blood pressure (DBP) ≥ 90 mmHg, or normal blood pressure with concomitant use of antihypertensive medications. Definite diabetes was defined as fasting plasma glucose (FG) ≥ 7.0 mmol/l or normal FG with concomitant use of insulin or oral hypoglycemic agents. CAD occurs when the arteries that supply blood to heart muscle become hardened and narrowed with calcified, non-calcified and mixed plaques. We defined coronary artery and branches having calcified, non-calcified and mixed plaques with plaques ≥ 70% stenosis, or ≥ 50% stenosis of the left main coronary artery as CAD of moderate to severe coronary atherosclerosis (CAD-msCA), the coronary plaques not CAD-msCA as CAD of minimal to mild CA (CAD-mmCA), and the overall as CAD-CA [23].

### Statistical Analysis

Descriptive statistics were calculated for all variables and significant differences in continuous variables were determined by ANOVA and LSD test was conducted to have Post-hoc multiple comparisons. The categorical percentile values were compared by Chi-squared test (χ^2^-test). The age and gender-adjusted univariate and multivariate logistic regression models were used to evaluate the association of body ectopic fat deposition with CAD-msCA or CAD-CA having plaques in coronary artery branches defined as above. All statistical analyses were performed using SPSS 17.0 software (SPSS Inc., Chicago, IL, USA) and a two-tailed P value < 0.05 was considered to be statistically significant.

## Results

### Age and gender difference of the distribution of body ectopic fat deposition in Chinese individuals

A total of 750 participants aged 50 years and over (378 males and 372 females), who completed CCTA scan and CT scan of the chest and abdomen, were given a detailed analysis of coronary artery atherosclerotic plaques and body ectopic fat distribution. The demographics and clinical characteristics of the patient population were described in Supplement Table 2 and the results of body fat with increasing age in patients of both genders were shown in Table 1.

**Table 1:** The ectopic fat distribution and coronary artery atherosclerosis in participants stratified by age and gender.

Age, years	50–59 (n = 175)	60–69 (n = 388)	70–79 (n = 161)	≥ 80 (n = 26)

**CAD-CA, % (n)**				
Male (378)	54.5 (55)	70.2 (134)**	80.0 (60)**	90.9 (10)**
Female (372)	27.0 (20)	41.6 (82)**	65.1 (56)**	80.0 (12)**
Total (750)	42.9 (75)	55.7 (216)**	72.0 (116)**	84.6 (22)**
**CAD-msCA, % (n)**				
Male (378)	20.8 (21)	34.0 (65)**	49.3 (37)**	54.5 (6)**
Female (372)	5.4 (4)	12.2 (24)*	16.3 (13)*	33.3 (5)*
Total (750)	14.2 (25)	22.9 (89)*	31.7 (51)**	42.3 (11)**
**CAC score**				
Male (378)	47.4 (13–81)	191.4 (117–265)**	355.7 (167–543)**	731.0 (131–1330)**
Female (372)	2.0 (0.6–4.6)	31.2 (15–46)*	159.7 (43–276)**	163.2 (18–344)**
Total (750)	28.2 (8.6–47)	110.0 (71–148)*	251.0 (143–358)**	403.3 (129–677)**
**BMI, kg/m^2^**				
Male (378)	25.3 (24.6–26.0)	25.2 (24.7–25.6)	24.6 (23.9–25.3)	23.1 (21.1–25.1)*
Female (372)	23.8 (23.0–24.6)	24.5 (24.0–24.9)	23.7 (23.0–24.5)	24.3 (22.4–26.2)
Total (750)	24.7 (24.1–25.8)	24.8 (24.5–25.1)	24.1 (23.6–24.6)	23.8 (22.5–25.1)
**WC, cm**				
Male (378)	92.5 (90.5–94.4)	91.0 (89.7–92.3)	89.5 (87.5–91.6)*	85.3 (77.9–92.7)
Female (372)	86.9 (84.9–88.8)	90.2 (88.8–91.6)*	91.1 (88.5–93.7)**	90.9 (86.1–95.6)
Total (750)	90.1 (88.6–91.5)	90.6 (89.7–91.5)	90.4 (88.7–92.1)	88.5 (84.5–92.5)
**VATA, cm^2^**				
Male (378)	145.0 (132–157)	146.0 (137–154)	139.2 (125–152)	115.2 (77–152)
Female (372)	100.5 (91–109)	111.8 (105–117)*	124.4 (115–133)**	136.6 (106–166)**
Total (750)	125.7 (117–134)	128.4 (122–133)	131.2 (123–139)	127.5 (102–152)
**SATA, cm^2^**				
Male (378)	164.0 (151–176)	158.5 (149–167)	148.3 (133–163)*	116.2 (77–155)
Female (372)	207.2 (179–235)	223.0 (211–234)	214.4 (196–232)	194.0 (153–234)
Total (750)	182.7 (168–197)	191.6 (183–199)	184.1 (171–197)	161.2 (128–194)
**VATV, cm^3^**				
Male (378)	2832 (2552–3112)	2688 (2501–2876)	2504 (2250–2759)	2177 (1201–3054)
Female (372)	1730 (1534–1926)	2128 (1981–2275)*	2208 (1966–2451)*	2170 (1815–2525)*
Total (750)	2354 (2156–2533)	2400 (2278–2521)	2344 (2169–2519)	2173 (1694–2652)
**SATV, cm^3^**				
Male (378)	2150 (1937–2363)	1911 (1778–2044)	1980 (1666–2294)	1345 (826–1863)
Female (372)	2392 (2087–2697)	2843 (2542–3144)	2456 (2218–2694)	1976 (1566–2386)
Total (750)	2255 (2077–2434)	2391 (2217–2566)	2238 (2043–2433)	1709 (1384–2034)
**LSR**				
Male (378)	1.22 (1.17–1.27)	1.21 (1.17–1.25)	1.27 (1.23–1.31)*	1.39 (1.30–1.49)
Female (372)	1.25 (1.19–1.30)	1.26 (1.23–1.30)	1.33 (1.28–1.38)*	1.29 (1.19–1.39)
Total (750)	1.23 (1.19–1.27)	1.24 (1.21–1.26)	1.30 (1.27–1.34)**	1.33 (1.26–1.40)*
**PAFV, cm^3^**				
Male (378)	22.2 (19.7–24.6)	23.0 (21.5–24.4)	21.8 (19.6–23.9)	20.2 (12.1–28.2)
Female (372)	10.3 (9.1–11.5)	12.5 (11.7–13.3)**	15.3 (13.9–16.6)**	15.2 (11.2–19.2)**
Total (750)	17.2 (15.4–18.9)	17.7 (16.7–18.6)	18.3 (17.0–19.6)	17.3 (13.4–21.2)
**EFV, cm^3^**				
Male (378)	64.3 (55.2–73.4)	59.2 (54.6–63.8)	53.8 (46.7–60.9)	53.9 (34.0–73.9)
Female (372)	29.3 (24.8–33.8)	36.5 (33.9–39.1)**	41.6 (37.3–46.0)**	51.3 (36.0–66.6)**
Total (750)	49.5 (43.4–55.6)	47.7 (44.9–50.5)	47.2 (43.1–51.4)	52.4 (41.8–63.7)
**PaCFV, cm^3^**				
Male (378)	83.8 (76.6–91.1)	80.5 (75.8–85.2)	79.2 (70.4–88.0)	74.6 (47.6–91.5)
Female (372)	68.5 (62.0–75.9)	76.6 (71.6–80.6)*	82.6 (75.4–89.9)**	94.6 (73.4–105.8)**
Total (750)	77.3 (72.0–82.6)	78.5 (75.0–81.5)	81.1 (75.5–86.6)	86.1 (70.2–92.1)

Data are presented as mean (95% confidence interval, CI) for continuous and % (numbers, n) for categorical characteristics. Abbreviations: CAD-CA, coronary artery atherosclerosis; CAD-msCA, CAD moderate to severe coronary atherosclerosis; CAC score, coronary artery calcium score; BMI, body mass index, kg/m^2^; WC, waist circumference, cm; VATA, visceral adipose tissue area, cm^2^; SATA, subcutaneous adipose tissue area, cm^2^; VATV, visceral adipose tissue volume, cm^3^; SATV, subcutaneous adipose tissue volume, cm^3^; LSR, liver-to-spleen attenuation ratio; PAFV, periaortic fat volume, cm^3^; EFV, epicardial fat volume, cm^3^; PaCFV, paracardial fat volume, cm^3^. * p < 0.05 and ** p < 0.01 compared with the group of aged 50–59 years old.

The proportion of participants having overall CAD-CA was 57.2%, with CAD-msCA in 23.5 %, and both proportions were significantly greater in men than in women (68.5% vs. 45.7% for CAD-CA and 34.1% vs. 12.6% for CAD-msCA, both p < 0.001). In parallel with CAD-CA, the CAC score were significantly higher in men than in women (201.2 vs. 60.4, p < 0.001). A significantly increased CAD-CA was seen in both genders with increasing age, with 54.5%, 70.2%, 80.0%, 90.9% in men and 27.0 %, 41.6%, 65.1%, 80.0% in women having CAD-CA, and 20.8%, 34.0%, 49.3%, 54.5% in men, and 5.4%, 12.2%, 16.3%, 33.3% in women having CAD-msCA in the 50–59, 60–69, 70–79 and over 80 years age groups, respectively.

Overall BMI was greater in men than women, and the male participants had higher occurrence of obesity than female participants, 15.4% vs. 11.0% (p = 0.002). There was no significant difference of WC between men and women (mean 90.9 cm in men and 89.8 cm in women, p = 0.105). However, significantly higher VAT measurements were observed in men than in women (143.4 cm^2^ vs. 113.6 cm^2^ for VATA and 2673 cm^3^ vs. 2070 cm^3^ for VATV in men and women, both p < 0.001), while women had significantly higher abdominal SAT than men (216.6 cm^2^ vs. 156.6 cm^2^ for SATA and 2628 cm^3^ vs. 1969 cm^3^ for SATV in men and women, both p < 0.001). As for liver fat, men had significantly lower LSR indicating higher liver adiposity than women (1.23 in men and 1.28 in women, p = 0.007). There was significantly higher PAFV and EFV in men than those values in women, 22.4 cm^3^ vs. 12.8 cm^3^ for PAFV and 59.4 cm^3^ vs. 36.8 cm^3^ for EFV in men and women (both p < 0.001), while no significant difference in PaCFV (p = 0.097) or the proportion with fatty liver (p = 0.181) were observed between men and women.

With increasing age, a significant decline in BMI and WC was observed in men (both P < 0.05), and there was no difference in BMI but a significant increase of WC found in women (P < 0.05). In parallel with the change of WC, a significantly increased VAT was observed in women (P < 0.01), and a significantly decreased abdominal SAT observed in men (P < 0.05) with increasing age. There was a significant decline of liver fat with increasing age in bother genders (P < 0.05), and a significant increase of PAFV, EFV and PaCFV was observed only in women (p < 0.01).

### Body ectopic fat depots comparing the individuals with and without coronary artery atherosclerosis

The mean CAC score was 229.7 in CAD-CA with a large variation (Table 2). It has been reported that CCTA improved risk stratification over coronary calcium scoring in symptomatic patients with suspected CAD [24] and coronary arteries having minimal to mild plaques also had clinical importance [25]. Therefore, we evaluated different types of coronary artery plaques and the results were shown in Table 2. We found a high proportion of non-calcified plaques in CAD-CA patients, with 55.3% in CAD-mmCA and 59.7% in CAD-msCA patients. The non-calcified plaques in CAD-msCA were coronary artery multiple type plaques containing non-calcified ones, 29.0% of three types of calcified, non-calcified and mixed plaques, 15.9% of calcified and non-calcified plaques, and 9.1% of exclusive non-calcified plaques, while non-calcified plaques in CAD-mmCA were mostly of exclusive non-calcified plaques (38.7%).

**Table 2:** Plaque analysis in coronary arteries and branches.

	CAD-CA	CAD-mmCA	CAD-msCA

% (n)	57.2 (429)	34.5 (259)	22.7 (170)
CAC score	229.7 (174–284)	27.9 (18.3–37.5)	519.9 (398–641)**
CAC score maximum		786	4176
Plaque types			
Plaques having non-calcified ones	57.1 (245)	55.3 (140)	59.7 (105)
Calcified plaques	24.2 (104)	28.5 (72)	18.2 (32)
Non-calcified plaques	26.6 (114)	38.7 (98)	9.1 (16)
Mixed plaques	6.1 (26)	8.3 (21)	2.8 (5)
Calcified and non-calcified plaques	11.4 (48)	7.9 (20)	15.9 (28)
Calcified and mixed plaques	12.6 (54)	7.9 (20)	19.3 (34)
Non-calcified and mixed plaques	5.2 (22)	4.7 (12)	5.7 (10)
All three types of plaques	14.5 (61)	4.0 (10)	29.0 (51)

Data are presented as mean (95% confidence interval, CI) for continuous and % (numbers, n) for categorical characteristics. Abbreviations: CAD-CA, coronary artery atherosclerosis; CAD-mmCA, CAD minimal to mild coronary atherosclerosis; CAD-msCA, CAD moderate to severe coronary atherosclerosis; CAC score, coronary artery calcium score. ** p < 0.01 compared with CAD-mmCA.

Since a high proportion of non-calcified plaques was observed in CAD-CA, which showed a zero CAC score, therefore, we used the coronary artery plaques measured from CCTA to define overall CAD-CA and CAD-msCA, and further to evaluate their associations with body ectopic fat depots. The ectopic fat distribution of participants was shown in Table 3, and in supplement Tables 3 and 4 which was stratified by gender. Individuals with CAD-CA had significantly increased BMI, WC, VAT, PAFV and EFV, but not abdominal SAT. In patients with CAD-CA, there was a significant increase of WC in women (p = 0.014), and a non-significant trend in men (p = 0.072), with no significant difference of BMI in both genders compared to those without CA. A significant increase of VAT (p = 0.008 for VATA and p < 0.001 for VATV), PAFV (p < 0.001), and EFV (p = 0.005) was observed in women, while a significant increase of liver fat (p = 0.049) and PAFV (p = 0.031), but no significant change of VAT was observed in men. There was no significant difference in abdominal SAT in both genders between participants with CA or without CA.

**Table 3:** The clinical characteristics and ectopic fat distribution of the participants.

	no CAD-CA (n = 321)	CAD-msCA/mmCA (n = 429)	p-value	No/CAD-mmCA (n = 574)	CAD-msCA (n = 176)	p-value

Gender, % men (n)	37.1 (119)	60.4 (259)	<0.001	43.4 (249)	73.3 (129)	<0.001
Age, years	62.7 (62.0–63.4)	66.3 (65.6–67.0)	<0.001	64.0 (63.4–64.6)	67.3 (66.2–68.4)	<0.001
CAD-CA, % (n)		57.2 (429)			23.5 (176)	
CAC score	0	229.7 (174–284)	–	12.3 (7.9–16.6)	519.9 (398–641)	<0.001
Overweight, % (n)	36.4 (117)	48.3 (48.3)	0.004	41.5 (238)	48.9 (86)	0.029
Obesity, % (n)	13.4 (43)	13.1 (56)	0.132	12.0 (69)	17.0 (30)	0.038
Diabetes, % (n)	13.4 (43)	26.3 (113)	<0.001	17.1 (98)	33.0 (58)	<0.001
Hypertension, % (n)	52.3 16)	71.3 (306)	<0.001	59.8 (343)	74.4 (131)	<0.001
Current smoking, % (n)	19.6 (63)	27.0 (116)	0.011	22.6 (130)	27.8 (49)	0.066
Statin use, % (n)	6.9 (22)	16.8 (72)	<0.001	9.9 (57)	21.0 (37)	<0.001
HTN med, % (n)	32.4 (104)	52.0 (223)	<0.001	38.7 (222)	59.7 (105)	<0.001
DM med, % (n)	4.7 (15)	14.0 (60)	<0.001	7.1 (41)	19.3 (34)	<0.001
SBP, mmHg	128.3 (126–130)	136.7 (135–138)	<0.001	131.5 (130–132)	138.3 (135–140)	<0.001
BMI, kg/m^2^	24.3 (23.9–24.7)	24.8 (24.4–25.1)	0.043	24.4 (24.1–24.6)	25.3 (24.8–25.8)	0.001
WC, cm	89.0 (87.9–90.1)	91.4 (90.5–92.3)	0.007	89.6 (88.8–90.5)	92.7 (91.4–94.0)	<0.001
**Ectopic fat deposition**						
VATA, cm^2^	120.0 (114–126)	134.4 (129–139)	<0.001	121.6 (117–125)	150.3 (141–158)	<0.001
SATA, cm^2^	191.7 (181–202)	183.2 (175–190)	0.180	187.3 (180–194)	185.2 (174–196)	<0.001
VATV, cm^3^	2171 (2042–2300)	2514 (2397–2630)	<0.001	2234 (2138–2329)	2807 (2618–2996)	0.776
SATV, cm^3^	2266 (2136–2397)	2327 (2165–2489)	0.585	2291 (2161–2422)	2335 (2152–2517)	<0.001
LSR	1.28 (1.25–1.31)	1.23 (1.21–1.25)	0.005	1.26 (1.24–1.28)	1.22 (1.19–1.26)	0.737
Fatty liver, % (n)	19.0 (61)	23.3 (100)	0.089	21.0 (119)	24.3 (42)	0.049
PAFV, cm^3^	15.0 (14.1–16.0)	19.7 (18.7–20.7)	<0.001	16.2 (15.5–17.0)	22.3 (20.6–24.0)	0.107
EFV, cm^3^	43.1 (39.8–46.5)	52.0 (49.0–54.9)	<0.001	44.7 (42.3–47.1)	59.5 (54.4–64.7)	<0.001
PaCFV, cm^3^	74.3 (70.6–78.0)	82.4 (79.1–85.6)	0.001	76.4 (73.7–79.1)	87.0 (81.6–92.5)	<0.001
**Laboratory parameters**						
FG, mmol/L	5.46 (5.30–5.62)	6.14 (5.91–6.36)	<0.001	5.63 (5.49–5.77)	6.55 (6.12–6.97)	<0.001
HbA1c, %	5.83 (5.75–5.92)	6.13 (6.04–6.23)	<0.001	5.90 (5.83–5.96)	6.36 (6.17–6.54)	<0.001
Triglyceride, mmol/L	1.84 (1.70–1.97)	2.02 (1.89–2.15)	0.058	1.92 (1.81–2.03)	2.02 (1.84–2.19)	0.386
HDL-C, mmol/L	1.43 (1.38–1.47)	1.32 (1.28–1.36)	<0.001	1.41 (1.37–1.44)	1.23 (1.18–1.29)	<0.001
LDL-C, mmol/L	2.94 (2.82–3.06)	3.10 (2.95–3.26)	0.095	3.14 (3.07–3.21)	3.14 (2.99–3.30)	0.973

Data are presented as mean (95 confidence interval, CI) for continuous and % (numbers, n) for categorical characteristics. Abbreviations: CAD-CA, coronary artery atherosclerosis; CAD-msCA, CAD moderate to severe coronary atherosclerosis; CAD-mmCA, CAD minimal to mild coronary atherosclerosis; CAC score, coronary artery calcium score; BMI, body mass index, kg/m^2^; HTN med, hypertension medication; DM med, diabetes medication; SBP, systolic blood pressure, mmHg; WC, waist circumference, cm; VATA, visceral adipose tissue area, cm^2^; SATA, subcutaneous adipose tissue area, cm^2^; VATV, visceral adipose tissue volume, cm^3^; SATV, subcutaneous adipose tissue volume, cm^3^; LSR, liver-to-spleen attenuation ratio; PAFV, periaortic fat volume, cm^3^; EFV, epicardial fat volume, cm^3^; PaCFV, paracardial fat volume, cm^3^; FG, fast glucose, mmol/L; HbA1c, hemoglobin A1c, %; HDL-C, high-density lipoprotein cholesterol, mmol/L; LDL-C, low-density lipoprotein cholesterol, mmol/L.

However, in patients with CAD-msCA compared to those with CAD-mmCA or no CA, there was no significant difference of BMI in both genders, and a significant increase of WC was observed in men (93.1 cm vs. 89.8 cm, p = 0.001), but this was not significant in women (91.5 cm vs. 89.5 cm, p = 0.222). In contrast to the change of WC, we observed an increase of VAT and PAFV in both men and women with more significant increase in women, for VAT (VATA, p = 0.002 in men and women; VATV, p = 0.021 in men and p < 0.001 in women) and PAFV (p = 0.014 in men and p < 0.001 in women). No significant difference in abdominal SAT and liver fat was observed in both genders between individuals with or without CAD-msCA.

These results suggested that there was a difference of body ectopic fat deposition between participants with CAD-CA and CAD-msCA, and that different localized ectopic fat depots might be associated with CAD-CA.

### Association of body ectopic fat deposition with coronary artery atherosclerosis

In this study the different body ectopic fat depots were measured in each participant at the same time and we found a strong correlation among different body fat depots (Table 4). In order to determine the association of body ectopic fat deposition with CAD-CA and/or CAD-msCA, a univariate model adjusted with age and gender and a multivariate logistic regression model were used and the results were shown in Table 5.

**Table 4:** Pearson correlation of body ectopic fat from multiple fat depots.

Pearson value	r*	p value	r**	p-value	r***	p-value

BMI, kg/m^2^	0.426	<0.001	0.564	<0.001	–0.287	<0.001
WC, cm	0.402	<0.001	0.655	<0.001	–0.243	<0.001
PAFV, cm^3^	1		0.563	<0.001	–0.219	<0.001
VATA, cm^2^	0.563	<0.001	1		–0.289	<0.001
VATV, cm^3^	0.597	<0.001	0.704	<0.001	–0.323	<0.001
LSR	–0.219	<0.001	–0.289	<0.001	1	
EFV, cm^3^	0.679	<0.001	0.580	<0.001	–0.186	<0.001

Abbreviations: BMI, body mass index, kg/m^2^; meds, medications; WC, waist circumference, cm; PAFV, periaortic fat volume, cm^3^; VATA, visceral adipose tissue area, cm^2^; LSR, liver-to-spleen attenuation ratio; EFV, epicardial fat volume, cm^3^. r* means the correlation coefficients between PAFV and other variables; r** means the correlation coefficients between VATA and other variables; r*** means the correlation coefficients between LSR and other variables.

**Table 5:** Multivariate association of body ectopic fat deposition and coronary artery atherosclerosis.

Variables	CAD-CA		CAD-msCA	

	OR (95% CI)	p-value	OR (95% CI)	p-value

**Univariate model adjusted with age and gender**				
BMI, kg/m^2^	1.05 (1.01–1.11)	0.133	1.09 (1.03–1.15)	0.001
WC, cm	1.02 (1.01–1.04)	0.003	1.03 (1.01–1.05)	<0.001
VATA, cm^2^	1.00 (1.00–1.01)	0.011	1.01 (1.01–1.02)	<0.001
SATA, cm^2^	1.00 (0.99–1.00)	0.191	1.00 (0.99–1.00)	0.177
VATV, cm^3^	1.00 (1.00–1.00)	0.028	1.00 (1.00–1.00)	<0.001
SATV, cm^3^	1.00 (1.00–1.00)	0.073	1.00 (1.00–1.00)	0.137
LSR	0.29 (0.15–0.56)	0.002	0.52 (0.25–1.01)	0.050
PAFV, cm^3^	1.05 (1.03–1.07)	0.001	1.06 (1.04–1.08)	<0.001
EFV, cm^3^	1.01 (1.00–1.02)	0.021	1.01 (1.01–1.02)	<0.001
PaCFV, cm^3^	1.00 (1.00–1.01)	0.044	1.01 (1.00–1.02)	<0.001
**Multivariate logistic regression model**				
WC, cm	1.02 (1.00–1.04)	0.039	1.02 (0.99–1.05)	0.146
VATA, cm^2^	1.00 (0.99–1.01)	0.904	1.00 (1.00–1.01)	0.003
SATA, cm^2^	1.00 (0.99–1.00)	0.609	1.00 (0.99–1.01)	0.101
VATV, cm^3^	1.00 (1.00–1.00)	0.307	1.00 (1.00–1.00)	0.017
SATV, cm^3^	1.00 (1.00–1.00)	0.105	1.00 (1.00–1.00)	0.229
LSR	0.44 (0.21–0.95)	0.036	0.83(0.35–1.94)	0.670
PAFV, cm^3^	1.02 (1.00–1.04)	0.028	1.02 (1.00–1.04)	0.024
EFV, cm^3^	1.00 (0.99–1.01)	0.286	1.01 (1.00–1.02)	0.022
PaCFV, cm^3^	1.00 (1.00–1.01)	0.074	1.00 (1.00–1.01)	0.014

Abbreviations: OR, odds ratio; CI, confidence interval; CAD-CA, coronary artery atherosclerosis; CAD-msCA, moderate to severe coronary artery atherosclerosis; WC, waist circumference, cm; VATA, visceral adipose tissue area, cm^2^; SATA, subcutaneous adipose tissue area, cm^2^; VATV, visceral adipose tissue volume, cm^3^; SATV, subcutaneous adipose tissue volume, cm^3^; LSR, liver-to-spleen attenuation ratio; PAFV, periaortic fat volume, cm^3^; EFV, epicardial fat volume, cm^3^; PaCFV, paracardial fat volume, cm^3^. Multivariate logistic regression model for body ectopic fat adjusted for age, gender, BMI, hypertension, diabetes, current smoking, TG, LDL-C and HDL-C.

In the univariate model adjusted with age and gender, we found a significant association of WC and body ectopic fat deposition with overall CAD-CA or CAD-msCA, except abdominal SAT. In multivariate logistic regression models, after adjustment for general adiposity with BMI and regular clinical CAD risk factors, we found a significant association of PAFV (p = 0.028) and liver fat (p = 0.036) with overall CAD-CA. However, for more severe CAD-msCA there were significant associations of PAFV (p = 0.024) and VAT (p = 0.003 for VATA and p = 0.017 for VATV). These results confirmed the association of different localized ectopic fat depots with CAD-CA and CAD-msCA.

Furthermore, we used quartile categorical multivariate logistic regression models of PAFV and VATA to look at the association of CAD and ectopic fat depots in detail (Table 6). The mean PAFV overall was 17.7 cm^3^ and the 25th, 50th and 75th percentile values were 10.3, 15.2 and 22.7 cm^3^, respectively. The prevalence of CAD-CA in the four PAFV quartiles was 71, 105, 119 and 134 patients, respectively, and the results of PAFV quartile categorical multivariate logistic regression models indicated a significant association of the upper three quartiles of PAFV with an increased prevalence of CAD-CA in models 1 and 2, and the highest quartile in model 3 after additional adjustment of abdominal obesity with WC. For CAD-msCA, the prevalence in the four PAFV quartiles was 16, 37, 53 and 70 patients, respectively, and the results of PAFV quartile categorical multivariate logistic regression models indicated a significant association of the upper three quartiles of PAFV with an increased prevalence of CAD-CA in model 1, and the highest quartile in models 2 and 3. The mean VATA overall was 128.3 cm^2^ and the 25th, 50th and 75th percentile values were 89.4, 121.7 and 156.8 cm^2^, respectively. The prevalence of CAD-msCA in the four VATA quartiles was 24, 31, 52 and 63 patients, respectively, and the results of VATA quartile categorical multivariate logistic regression models indicated a significant association of the upper two quartiles of VATA with an increased prevalence of CAD-msCA in models 1, 2 and 3, while only the upper 2 quartiles of VATA were associated with CAD-CA in model 1 and no association was observed in models 2 and 3.

**Table 6:** CT-derived PAFV/VATA and coronary atherosclerosis in Chinese middle aged and elderly patients.

PAFV and CAD-CA	CAD-CA Cases/Total	Prevalence of CAD-CA, OR (95% CI)		

		Model 1	Model 2	Model 3

Logistic Regression	429/750	1.03 (1.01–1.05)**	1.02 (1.00–1.04)*	1.02 (1.00–1.04)
Per quartile*				
Q1	71/188	Reference	Reference	Reference
Q2	105/187	1.76 (1.13–2.71)^	1.68 (1.03–2.65)^	1.33 (0.93–2.49)
Q3	119/187	1.93 (1.22–3.04)^^	1.74 (1.06–2.87)^	1.65 (1.00–2.73)
Q4	134/188	2.21 (1.33–3.68)^^	1.90 (1.07–3.37)^	1.73 (1.00–3.10)^
	**CADmsCA Cases/Total**	**Prevalence of CAD-msCA, OR (95% CI)**		

		**Model 1**	**Model 2**	**Model 3**

Logistic Regression	176/750	1.02 (1.01–1.04)	1.02 (1.00–1.04)	1.01 (0.99–1.03)
Per quartile*				
Q1	16/188	Reference	Reference	Reference
Q2	37/187	2.19 (1.14–4.20)^	2.01 (1.03–3.93)^	1.98 (0.99–3.86)
Q3	53/187	2.77 (1.47–5.23)^^	2.18 (1.08–4.40)^	2.03 (1.00–4.12)
Q4	70/188	3.15 (1.64–6.05)^^	2.26 (1.17–4.42)^	2.20 (1.13–4.29)^
**VATA and CAD**	**CAD-CA Cases/Total**	**Prevalence of CAD-CA, OR (95% CI)**		

		**Model 1**	**Model 2**	**Model 3**

Logistic Regression	429/750	1.00 (1.00–1.01)^	1.00 (0.99–1.01)	1.00 (0.99–1.01)
Per quartile*				
Q1	84/188	Reference	Reference	Reference
Q2	97/187	1.33 (0.86–2.06)	1.13 (0.70–1.84)	1.07 (0.66–1.74)
Q3	129/187	2.27 (1.64–3.84)^^	1.96 (1.00–3.04)	1.73 (0.98–3.00)
Q4	119/188	1.90 (1.01–2.85)^	1.75 (0.98–2.50)	1.30 (0.84–2.04)
	**CADmsCA Cases/Total**	**Incidence of CAD-msCA, OR (95% CI)**		

		**Model 1**	**Model 2**	**Model 3**

Logistic Regression	176/750	1.01 (1.00–1.02)^^	1.01 (1.00–1.02)^	1.00 (1.00–1.01)^
Per quartile*				
Q1	23/188	Reference	Reference	Reference
Q2	34/187	1.63 (0.88–2.98)	1.29 (0.67–2.47)	1.26 (0.65–2.43)
Q3	53/187	2.72 (1.54–4.81)^^	1.99 (1.04–3.79)^	1.94 (1.00–3.75)^
Q4	67/188	3.04 (1.74–5.32)^^	2.23 (1.14–4.39)^	2.07 (1.01–4.27)^

**Model 1**: Adjusted for age and gender. **Model 2**: As for model 1, additionally adjusted for BMI, current smoking, hypertension, diabetes, plasma low-density lipoprotein cholesterol and high-density lipoprotein cholesterol. **Model 3**: As for model 2, additionally adjusted for waist circumference. * The ranges of PAFV per quartile were as follows: Q1: 4.2–10.3cm^3^. Q2: 10.4–15.2 cm^3^. Q3: 15.3–22.7 cm^3^. Q4: 22.8–51.5 cm^3^, and VATA Q1: 21.8–89.2 cm^3^. Q2: 89.4–121.4 cm^3^. Q3: 121.7–156.6 cm^3^. Q4: 156.8–298.8 cm^3^. Abbreviations: CT, computed tomography; CAD-CA, coronary artery atherosclerosis; CAD-msCA, moderate to severe coronary artery atherosclerosis; OR, odd ratio; CI, confidence interval. ^: p < 0.05, ^^: p < 0.01 in logistic regression analysis.

These results suggested that PAFV was associated with an increased prevalence of CAD-CA and CAD-msCA in model 2 that was independent of clinical risk factors and general obesity, and in model 3 independent of abdominal obesity in patients of the highest PAFV quartile, while VATA was associated with an increased prevalence of CAD-msCA in the patients from the upper 2 VATA quartiles that were independent of clinical risk factors and general and abdominal obesity.

## Discussion

There are several limitations in this study which deserve comment. The study subjects are middle aged and elderly Chinese with relatively few females with CAD-msCA and it is an observational cross-sectional study which can only demonstrate associations with CA and it cannot prove that PAFV or VAT is a causative risk factor for CA. Also we used CT angiography in this study to measure the coronary artery atherosclerotic plaques as the evaluation for CA and CT to quantify the body ectopic fat depots. Ideally more female CA and especially CAD-msCA patients need to be recruited and more accurate magnetic resonance quantification of ectopic fat depots should be performed to validate the accurate association.

The strengths of this study are that it examines a reasonably large group of middle aged and elderly Chinese subjects, which has not been studied in this detail before, and there are a similar number of male and female participants overall, which facilitates the identification of gender differences that are clearly present. There is also a reasonably wide age range among the subjects, although the number of subjects in certain age and gender groups becomes too small to provide definitive conclusions regarding the interaction of age and gender.

Obesity is associated with increased CV morbidity and mortality. However, not all overweight or obese individuals experience CV events [26], particularly among older adults [27]. In this study, we recruited middle aged and elderly Chinese outpatients to quantify the coronary atherosclerotic plaques from CCTA and measure the body ectopic fat from multiple fat depots at the same time from CT scan of the chest and abdomen for further evaluation of the associations. The main findings were age and gender differences of the distribution of body ectopic fat deposition in the middle aged and elderly Chinese patients. PAFV was significantly associated with overall CAD-CA and severe CAD-msCA, while VAT was associated with CAD-msCA and liver fat with overall CAD-CA even after adjustment for general adiposity BMI and clinical CAD risk factors. Further categorical multivariate logistic regression models showed a significant association of PAFV with CAD-CA and CAD-msCA that was independent of general obesity and clinical risk factors, and the highest PAFV quartile patients independent of abdominal obesity, while VATA was associated with an increased prevalence of CAD-msCA in the patients from the upper 2 VATA quartiles that were independent of clinic risk factors and general and abdominal obesity. These findings suggest that excess accumulation of periaortic fat is strongly associated with CA, and abdominal visceral fat deposition associated with more severe CAD-msCA, especially in the patients in the higher PAFV and VATA quartile. Therefore, measurement of body ectopic fat deposition may provide additional information regarding its association with CA especially more severe CAD-msCA.

Ethnic differences of fat deposition were reported in the multi-ethnic Southall And Brent REvisited (SABRE) study and increased overall truncal fat contributed to the excess of diabetes in South Asian groups, particularly for women [12]. Compared to the above SABRE study [12], we found that the average BMI and WC was 25.0 kg/m^2^ and 90.9 cm for men, and 24.2 kg/m^2^ and 89.8 cm for women in our study, which is the same as the South Asian men, 25 kg/m^2^ and 91 cm, but less than the BMI of 26 kg/m^2^, and higher than the WC of 79 cm for South Asian women, and the VATA in our study was less in men but higher in women than in the South Asians.

Findings from epidemiological studies over the past 30 years identified that VAT, accurately measured by CT or MRI, is an independent risk marker of cardiovascular and metabolic morbidity and mortality [9,28,29]. VAT was a stronger correlate of most metabolic risk factors than pericardial fat in the Framingham Heart Study [6]. Moreover a recent study showed that body ectopic fat distribution was useful for evaluating risk and prognosis of CAD, and higher VAT and lower SAT were correlated with the extent and severity of coronary artery plaques [30]. In the present study we confirmed the abdominal fat deposition as a significant association risk for severe CAD-msCA in the Chinese middle aged and elderly patients.

In addition to abdominal obesity, fat accumulation around the aorta and heart may impact CV health. Excess fat accumulation around the proximal aorta (i.e. periaortic fat) and the heart (i.e. pericardial fat) may have more adverse effects on CV health given their anatomic location [31]. These fat depots may impact adjacent tissues and organs through locally secreted biochemical factors that adversely affect neighboring cardiomyocytes and vascular endothelial and smooth muscle cells [31,32,33]. A previous study showed that older adults with a high risk for CV events have greater periaortic fat than low-risk adults, even after accounting for BMI [34]. Moreover, in 1205 participants from the Framingham Heart Study Offspring cohort, periaortic fat was associated with the clinical spectrum of atherosclerosis from low ankle-brachial index to peripheral arterial diseases in multivariable logistic regression after adjusting for BMI or VAT [10]. In this study we found that PAFV was significantly associated with overall CAD-CA, as well as more severe CAD-msCA, in the Chinese middle aged and elderly patients even after the adjustment with the abdominal obesity marker WC in the patients of the highest PAFV quantile.

A prospective study from the Framingham Heart Study Offspring cohort reported that VAT and periaortic fat, but not pericardial fat and SAT, were associated with the CVD events [9]. In this study we did not find an association of EFV with coronary atherosclerosis in the multivariate logistic regression model after adjustment. Epicardial fat is a unique fat compartment between the myocardium and the visceral pericardium sharing a common embryologic origin with the visceral fat depot. Epicardial adipose tissue was considered as a source of inflammatory mediators that might directly influence the myocardium and coronary arteries leading to the development and progression of coronary atherosclerosis [33]. Quantification of epicardial adipose tissue volume by CT imaging has been independently associated with coronary plaque burden, plaque composition and vulnerability [35], the development of coronary atherosclerosis in healthy subjects and the risk of future coronary events in patients with CAD [36]. In a study of ethnic Chinese in Taiwan, measurement of epicardial adipose tissue thickness in the left atrioventricular groove was reported to be a more accurate assessment of metabolic risk than anthropometric indexes and VAT [37]. However, recent studies did not find an association of pericardial adipose tissue and CVD after adjustment for the risk covariates [38], and in this study of Chinese middle aged and elderly patients we did not observe a significant association of epicardial or paracardial fat and the presence of CAD-CA either.

In conclusion, we observed age and gender differences of body ectopic fat distribution in Chinese patients with higher VAT and PAFV in men and higher SAT in women. There was a decline of SAT in men and an increase of VAT and PAFV in women with increasing age. PAFV was significantly associated with overall CAD-CA and severe CAD-msCA, and VAT was associated with CAD-msCA. It would be useful to examine these associations in a longitudinal study to determine if the ectopic fat depots have any relationship to CV outcomes and to assess if certain interventions might have a selective effect on particular fat depots. The findings of our study suggest that CCTA measurement of the ectopic body fat may provide important insights into coronary atherosclerosis especially in severe atherosclerosis, thus it may be worthwhile to use these measurements in future clinical practice to further establish whether they have implications for prognosis or interventions to improve CV outcomes.

## Additional File

The additional file for this article can be found as follows:

10.5334/gh.1078.s1 Supplement File. Supplement Tables 1–4 and Supplement Figure 1.

## References

[1] Afshin A, Forouzanfar MH, Reitsma MB, S, et al. Health effects of overweight and obesity in 195 countries over 25 years. N Engl J Med. 2017; 377: 13–27. DOI: 10.1056/NEJMoa1614362 28604169 PMC5477817

[2] Chen Y, Peng Q, Yang Y, Zheng S, Wang Y, Lu W. The prevalence and increasing trends of overweight, general obesity, and abdominal obesity among chinese adults: A repeated cross-sectional study. BMC Public Health. 2019; 19: 1293. DOI: 10.1186/s12889-019-7633-0 31615464 PMC6794823

[3] Tchernof A, Despres JP. Pathophysiology of human visceral obesity: An update. Physiol Rev. 2013; 93: 359–404. DOI: 10.1152/physrev.00033.2011 23303913

[4] Gonzalez-Muniesa P, Martinez-Gonzalez MA, Hu FB, et al. Obesity. Nat Rev Dis Primers. 2017; 3: 17034. DOI: 10.1038/nrdp.2017.34 28617414

[5] Sahakyan KR, Somers VK, Rodriguez-Escudero JP, et al. Normal-weight central obesity: Implications for total and cardiovascular mortality. Ann Intern Med. 2015; 163: 827–835. DOI: 10.7326/M14-2525 26551006 PMC4995595

[6] Rosito GA, Massaro JM, Hoffmann U, et al. Pericardial fat, visceral abdominal fat, cardiovascular disease risk factors, and vascular calcification in a community-based sample: The framingham heart study. Circulation. 2008; 117: 605–613. DOI: 10.1161/CIRCULATIONAHA.107.743062 18212276

[7] Mahabadi AA, Massaro JM, Rosito GA, et al. Association of pericardial fat, intrathoracic fat, and visceral abdominal fat with cardiovascular disease burden: The framingham heart study. Eur Heart J. 2009; 30: 850–856. DOI: 10.1093/eurheartj/ehn573 19136488 PMC3693564

[8] Zhang C, Rexrode KM, van Dam RM, Li TY, Hu FB. Abdominal obesity and the risk of all-cause, cardiovascular, and cancer mortality: Sixteen years of follow-up in us women. Circulation. 2008; 117: 1658–1667. DOI: 10.1161/CIRCULATIONAHA.107.739714 18362231

[9] Britton KA, Massaro JM, Murabito JM, Kreger BE, Hoffmann U, Fox CS. Body fat distribution, incident cardiovascular disease, cancer, and all-cause mortality. Journal of the American College of Cardiology. 2013; 62: 921–925. DOI: 10.1016/j.jacc.2013.06.027 23850922 PMC4142485

[10] Fox CS, Massaro JM, Schlett CL, et al. Periaortic fat deposition is associated with peripheral arterial disease: The framingham heart study. Circulation. Cardiovascular imaging. 2010; 3: 515–519. DOI: 10.1161/CIRCIMAGING.110.958884 20639302 PMC3060043

[11] Wassel CL, Laughlin GA, Araneta MR, et al. Associations of pericardial and intrathoracic fat with coronary calcium presence and progression in a multiethnic study. Obesity (Silver Spring). 2013; 21: 1704–1712. DOI: 10.1002/oby.20111 23666866 PMC3748173

[12] Eastwood SV, Tillin T, Dehbi HM, et al. Ethnic differences in associations between fat deposition and incident diabetes and underlying mechanisms: The sabre study. Obesity (Silver Spring). 2015; 23: 699–706. DOI: 10.1002/oby.20997 25645144 PMC4463764

[13] Lim U, Monroe KR, Buchthal S, et al. Propensity for intra-abdominal and hepatic adiposity varies among ethnic groups. Gastroenterology. 2019; 156: 966–975.e910. DOI: 10.1053/j.gastro.2018.11.021 30445012 PMC6409195

[14] Jiang B, Chen Y, Zhou K, et al. Comparison of abdominal obesity and fatty liver and their association with insulin resistance and metabolic syndrome in chinese adults. 2019; 27: 707–715. DOI: 10.1002/oby.22432 30942551

[15] Huo L, Li K, Deng W, et al. Optimal cut-points of visceral adipose tissue areas for cardiometabolic risk factors in a chinese population: A cross-sectional study. Diabet Med. 2019; 36: 1268–1275. DOI: 10.1111/dme.14060 31257674

[16] Ei Ei Khaing N, Shyong TE, Lee J, Soekojo CY, Ng A, Van Dam RM. Epicardial and visceral adipose tissue in relation to subclinical atherosclerosis in a chinese population. PLoS One. 2018; 13: e0196328. DOI: 10.1371/journal.pone.0196328 29694442 PMC5919010

[17] Yip W, Mahal A. The health care systems of china and india: Performance and future challenges. Health Aff (Millwood). 2008; 27: 921–932. DOI: 10.1377/hlthaff.27.4.921 18607024

[18] Fan H, Li X, Zheng L, et al. Abdominal obesity is strongly associated with cardiovascular disease and its risk factors in elderly and very elderly community-dwelling chinese. Sci Rep. 2016; 6: 21521. DOI: 10.1038/srep21521 26882876 PMC4756331

[19] Kodama Y, Ng CS, Wu TT, et al. Comparison of ct methods for determining the fat content of the liver. AJR. American journal of roentgenology. 2007; 188: 1307–1312. DOI: 10.2214/AJR.06.0992 17449775

[20] Austen WG, Edwards JE, Frye RL, et al. A reporting system on patients evaluated for coronary artery disease. Report of the ad hoc committee for grading of coronary artery disease, council on cardiovascular surgery, american heart association. Circulation. 1975; 51: 5–40. DOI: 10.1161/01.CIR.51.4.5 1116248

[21] Puchner SB, Liu T, Mayrhofer T, et al. High-risk plaque detected on coronary ct angiography predicts acute coronary syndromes independent of significant stenosis in acute chest pain: Results from the romicat-ii trial. Journal of the American College of Cardiology. 2014; 64: 684–692. DOI: 10.1016/j.jacc.2014.05.039 25125300 PMC4135448

[22] Chen C, Lu FC. The guidelines for prevention and control of overweight and obesity in chinese adults. Biomed Environ Sci. 2004; 17 Suppl: 1–36. 15807475

[23] Cury RC, Abbara S, Achenbach S, et al. Cad-rads(tm) coronary artery disease – reporting and data system. An expert consensus document of the society of cardiovascular computed tomography (scct), the american college of radiology (acr) and the north american society for cardiovascular imaging (nasci). Endorsed by the american college of cardiology. J Cardiovasc Comput Tomogr. 2016; 10: 269–281. DOI: 10.1016/j.jcct.2016.04.005 27318587

[24] Al-Mallah MH, Qureshi W, Lin FY, et al. Does coronary ct angiography improve risk stratification over coronary calcium scoring in symptomatic patients with suspected coronary artery disease? Results from the prospective multicenter international confirm registry. European heart journal cardiovascular Imaging. 2014; 15: 267–274. DOI: 10.1093/ehjci/jet148 23966421

[25] Maddox TM, Stanislawski MA, Grunwald GK, et al. Nonobstructive coronary artery disease and risk of myocardial infarction. Jama. 2014; 312: 1754–1763. DOI: 10.1001/jama.2014.14681 25369489 PMC4893304

[26] Voulgari C, Tentolouris N, Dilaveris P, Tousoulis D, Katsilambros N, Stefanadis C. Increased heart failure risk in normal-weight people with metabolic syndrome compared with metabolically healthy obese individuals. Journal of the American College of Cardiology. 2011; 58: 1343–1350. DOI: 10.1016/j.jacc.2011.04.047 21920263

[27] Alam I, Ng TP, Larbi A. Does inflammation determine whether obesity is metabolically healthy or unhealthy? The aging perspective. Mediators of inflammation. 2012; 2012: 456456. DOI: 10.1155/2012/456456 23091306 PMC3471463

[28] Neeland IJ, Ross R, Despres JP, et al. Visceral and ectopic fat, atherosclerosis, and cardiometabolic disease: A position statement. Lancet Diabetes Endocrinol. 2019; 7: 715–725. DOI: 10.1016/S2213-8587(19)30084-1 31301983

[29] Fox CS, Massaro JM, Hoffmann U, et al. Abdominal visceral and subcutaneous adipose tissue compartments: Association with metabolic risk factors in the framingham heart study. Circulation. 2007; 116: 39–48. DOI: 10.1161/CIRCULATIONAHA.106.675355 17576866

[30] Tanaka T, Kishi S, Ninomiya K, et al. Impact of abdominal fat distribution, visceral fat, and subcutaneous fat on coronary plaque scores assessed by 320-row computed tomography coronary angiography. Atherosclerosis. 2019; 287: 155–161. DOI: 10.1016/j.atherosclerosis.2019.06.910 31295672

[31] Thalmann S, Meier CA. Local adipose tissue depots as cardiovascular risk factors. Cardiovascular research. 2007; 75: 690–701. DOI: 10.1016/j.cardiores.2007.03.008 17412312

[32] Nosalski R, Guzik TJ. Perivascular adipose tissue inflammation in vascular disease. British journal of pharmacology. 2017; 174: 3496–3513. DOI: 10.1111/bph.13705 28063251 PMC5610164

[33] Mazurek T, Zhang L, Zalewski A, et al. Human epicardial adipose tissue is a source of inflammatory mediators. Circulation. 2003; 108: 2460–2466. DOI: 10.1161/01.CIR.0000099542.57313.C5 14581396

[34] Brinkley TE, Leng X, Chughtai HL, et al. Periaortic fat and cardiovascular risk: A comparison of high-risk older adults and age-matched healthy controls. International journal of obesity. 2014; 38: 1397–1402. DOI: 10.1038/ijo.2014.29 24525960 PMC4143481

[35] Hassan M, Said K, Rizk H, et al. Segmental peri-coronary epicardial adipose tissue volume and coronary plaque characteristics. European heart journal cardiovascular Imaging. 2016; 17: 1169–1177. DOI: 10.1093/ehjci/jev298 26590399

[36] Kunita E, Yamamoto H, Kitagawa T, et al. Prognostic value of coronary artery calcium and epicardial adipose tissue assessed by non-contrast cardiac computed tomography. Atherosclerosis. 2014; 233: 447–453. DOI: 10.1016/j.atherosclerosis.2014.01.038 24530777

[37] Wang TD, Lee WJ, Shih FY, et al. Relations of epicardial adipose tissue measured by multidetector computed tomography to components of the metabolic syndrome are region-specific and independent of anthropometric indexes and intraabdominal visceral fat. The Journal of clinical endocrinology and metabolism. 2009; 94: 662–669. DOI: 10.1210/jc.2008-0834 19050055

[38] Chistiakov DA, Grechko AV, Myasoedova VA, Melnichenko AA, Orekhov AN. Impact of the cardiovascular system-associated adipose tissue on atherosclerotic pathology. Atherosclerosis. 2017; 263: 361–368. DOI: 10.1016/j.atherosclerosis.2017.06.017 28629772

