# Supplementary material for: Association of Periaortic Fat and Abdominal Visceral Fat with Coronary Artery Atherosclerosis in Chinese Middle Aged and Elderly Patients Undergoing Computed Tomography Coronary Angiography
Source: Glob Heart. 2021 Oct 19;16(1):74. doi: 10.5334/gh.1078 (PMC8533656; doi:10.5334/gh.1078)
Supplement: Supplement File. — Supplement Tables 1–4 and Supplement Figure 1. [file gh-16-1-1078-s1.pdf]

Supplement

Supplement Table 1: Intraclass correlation of inter-observer agreement for body fat measurement.

Parameter	ICC
VATA, cm^2	0.968
SATA, cm^2	1
VATV, cm^3	1
SATV, cm^3	1
PAFV, cm^3	0.998
EFV, cm^3	0.999
PaCFV, cm^3	0.999

Abbreviations: ICC, intraclass correlation; VATA, visceral adipose tissue area, cm^2; SATA, subcutaneous adipose tissue area, cm^2; VATV, visceral adipose tissue volume, cm^3; SATV, subcutaneous adipose tissue volume, cm^3; PAFV, periaortic fat volume, cm^3; EFV, epicardial fat volume, cm^3; PaCFV, paracardial fat volume, cm^3.

Supplement Table 2: The clinical characteristics and ectopic fat distribution of participants aged 50 years and over.

Age	All	Men	Women	p-value
	n=750	n=378	n=372	
Gender, %		50.4	49.6	
Age, years	64.8 (64.2-65.3)	64.2 (63.5-65.0)	65.3 (64.6-66.0)	0.057
CAD, % (n)	57.2 (429)	68.5 (259)	45.7 (170)	< 0.001
Obstructive CAD, % (n)	22.7 (170)	32.3 (122)	12.9 (48)	< 0.001
CAC score	131.4 (98.9-163.8)	201.2 (144.4-258.0)	60.4 (31.2-89.7)	< 0.001
Overweight, % (n)	43.3 (324)	47.2 (178)	39.2 (146)	0.001
Obesity, % (n)	13.2 (99)	15.4 (58)	11.0 (41)	0.002
Diabetes, % (n)	20.8 (156)	23.8 (90)	17.7 (66)	0.025
Hypertension, % (n)	63.2 (474)	65.3 (247)	61.0 (227)	0.227
Current smoking, % (n)	23.9 (179)	44.7 (169)	2.7 (10)	< 0.001
Statin, % (n)	12.5 (94)	13.8 (52)	11.3 (42)	0.323
HTN med, % (n)	43.6 (327)	46.6 (176)	40.6 (151)	0.106
DM med, % (n)	10.0 (75)	13.2 (50)	6.7 (25)	0.003
BMI, kg/m^2	24.6 (24.2-24.8)	25.0 (24.7-25.8)	24.2 (23.8-24.5)	< 0.001
SBP, mmHg	133.1 (131.9-134.3)	132.5 (131.0-134.1)	133.6 (131.8-135.5)	0.373
**Ectopic fat deposition**				
WC, cm	90.4 (89.7-91.1)	90.9 (90.0-91.9)	89.8 (88.7-90.8)	0.105
VATA, cm^2	128.3 (124.4-132.3)	143.4 (137.2-149.6)	113.6 (109.1-118.0)	< 0.001
SATA, cm^2	186.8 (180.6-193.0)	156.6 (149.9-163.3)	216.6 (207.2-226.0)	< 0.001
VATV, cm^3	2369 (2281-2456)	2673 (2540-2806)	2070 (1964-2176)	< 0.001
SATV, cm^3	2302 (2193-2410)	1969 (1860-2077)	2628 (2447-2808)	< 0.001
LSR	1.25 (1.24-1.27)	1.23 (1.20-1.26)	1.28 (1.25-1.30)	0.007
Fatty liver, % (n)	21.5 (161)	23.5 (89)	19.4 (72)	0.095
PAFV, cm^3	17.7 (17.0-18.4)	22.4 (21.4-23.5)	12.8 (12.2-13.4)	< 0.001
EFV, cm^3	48.2 (45.9-50.4)	59.4 (55.7-63.0)	36.8 (34.8-38.9)	< 0.001
PaCFV, cm^3	78.9 (76.4-81.4)	81.0 (77.4-84.5)	76.8 (73.4-80.2)	0.097
**Laboratory parameters**				
FG, mmol/L	5.85 (5.70-5.00)	5.95 (5.73-6.17)	5.75 (5.55-5.94)	0.176
HbA1c, %	6.00 (5.94-6.07)	6.04 (5.94-6.17)	5.96 (5.55-5.94)	0.238
Triglyceride, mmol/L	1.94 (1.85-2.03)	2.10 (1.95-2.26)	1.78 (1.67-1.88)	0.001
HDL-C, mmol/L	1.37 (1.34-1.40)	1.22 (1.18-1.25)	1.52 (1.48-1.58)	< 0.001
LDL-C, mmol/L	3.14 (3.07-3.21)	3.03 (2.93-3.12)	3.26 (3.16-3.35)	0.001

Data are presented as mean (95% confidence interval, CI) for continuous and % (numbers, n) for categorical characteristics. Abbreviations: CAD, coronary artery disease; CAC score, coronary artery calcium score; BMI, body mass index, kg/m^2; meds, medications; SBP, systolic blood pressure, mmHg; WC, waist circumference, cm; VATA, visceral adipose tissue area, cm^2; SATA, subcutaneous adipose tissue area, cm^2; VATV, visceral adipose tissue volume, cm^3; SATV, subcutaneous adipose tissue volume, cm^3; LSR, liver-to-spleen attenuation ratio; PAFV, periaortic fat volume, cm^3; EFV, epicardial fat volume, cm^3; PaCFV, paracardial fat volume, cm^3; FG, fast glucose, mmol/L; HbA1c, hemoglobin A1c, %; HDL-C, high-density lipoprotein cholesterol, mmol/L; LDL-C, low-density lipoprotein cholesterol, mmol/L.

Supplement Table 3: Body ectopic fat deposition in men and women with or without coronary artery disease.

	Men 378			Women 372		
	**no CAD-CA (n=119)**	**CADmsCA/mmCA (n=259)**	**p-value**	**no CAD-CA (n=202)**	**CAD msCA/mmCA (n=170)**	**p-value**
Age, years	61.6 (60.4-62.9)	65.5 (64.7-66.4)	<0.001	63.4 (62.5-64.2)	67.5 (66.5-68.6)	<0.001
CAD, % (n)		68.6 (259)			45.7 (170)	
Obstructive CAD, % (n)		34.1 (129)			12.6 (47)	
CAC score	0	293.7 (213-374)	<0.001	0	132.2 (69-194)	<0.001
Overweight, % (n)	40.3 (48)	50.2 (130)	0.075	34.1 (69)	45.3 (77)	0.041
Obesity, % (n)	15.9 (19)	15.1 (39)	0.335	11.9 (24)	10.0 (17)	0.247
Diabetes, % (n)	16.8 (20)	27.0 (70)	0.019	11.4 (23)	25.3 (43)	<0.001
Hypertension, % (n)	52.9 (63)	71.0 (184)	0.001	52.0 (105)	71.8 (122)	<0.001
Smokers, % (n)	47.9 (57)	43.2 (112)	0.231	3.0 (6)	2.3 (4)	0.486
Statin use, % (n)	6.7 (8)	17.0 (44)	0.004	6.9 (14)	16.5 (28)	0.003
HTN med, % (n)	32.8 (39)	52.9 (137)	<0.001	32.2 (65)	50.6 (86)	<0.001
DM med, % (n)	8.4 (10)	15.4 (40)	0.040	2.5 (5)	11.8 (20)	<0.001
SBP, mmHg	126.7 (124-129)	135.2 (133-137)	<0.001	129.2 (126-131)	138.9 (136-141)	<0.001
BMI, kg/m^2	24.8 (24.2-25.4)	25.1 (24.8-25.5)	0.380	24.0 (23.5-24 5)	24.3 (23.8-24.8)	0.406
WC, cm	89.6 (87.9-91.4)	91.5 (90.4-92.7)	0.072	88.6 (87.2-90.0)	91.2 (89.6-92.8)	0.014
**Ectopic fat deposition**						
VATA, cm^2	141.6 (130-152)	144.2 (1436-151)	0.710	107.9 (101-114)	120.1 (113-126)	0.008
SATA, cm^2	153.7 (141-165)	157.9 (149-166)	0.567	213.0 (198-227)	220.9 (209-232)	0.409
VATV, cm^3	2663 (2425-2900)	2678 (2517-2839)	0.918	1897 (1759-2035)	2271 (2112-2431)	<0.001
SATV, cm^3	1829 (1660-1999)	2030 (1893-2167)	0.093	2508 (2337-2680)	2767 (2429-3105)	0.160
LSR	1.27 (1.21-1.32)	1.21 (1.18-1.22)	0.049	1.29 (1.26-1.32)	1.26 (1.23-1.30)	0.199
Fatty liver, % (n)	25.2 (30)	22.8 (59)	0.337	15.3 (31)	24.1 (41)	0.021
PAFV, cm^3	20.7 (18.9-22.5)	23.3 (21.9-24.6)	0.031	11.7 (10.9-12.4)	14.2 (13.2-15.1)	<0.001
EFV, cm^3	58.5 (51.5-65.6)	59.7 (55.5-64.0)	0.765	34.1 (31.4-36.9)	40.1 (37.1-43.1)	0.005
PaCFV, cm^3	77.6 (71.6-83.6)	82.5 (78.1-86.9)	0.214	72.3 (67.6-77.1)	82.2 (77.5-86.9)	0.004
**Laboratory parameters**						
FG, mmol/L	5.45 (5.17-5.74)	6.17 (5.88-6.46)	0.003	5.46 (5.26-5.66)	6.08 (5.73-6.43)	0.002
HbA1c, %	5.76 (5.58-5.93)	6.17 (6.04-6.31)	<0.001	5.88 (5.78-5.97)	6.07 (5.94-6.20)	0.017
Triglyceride, mmol/L	2.04 (1.76-2.33)	2.13 (1.95-2.31)	0.614	1.71 (1.57-1.86)	1.85 (1.69-2.01)	0.210
HDL-C, mmol/L	1.24 (1.17-1.31)	1.20 (1.16-1.25)	0.355	1.54 (1.48-1.59)	1.50 (1.43-1.56)	0.345
LDL-C, mmol/L	2.97 (2.87-3.08)	3.14 (2.94-3.33)	0.101	2.91 (2.78-3.05)	3.08 (2.96-3.20))	0.112

Data are presented as mean (95% confidence interval, CI) for continuous and % (numbers, n) for categorical characteristics.

Abbreviations: CAD-CA, coronary artery atherosclerosis; CAD-msCA, CAD moderate to severe coronary atherosclerosis; CAD-mmCA, CAD minimal to mild coronary atherosclerosis; BMI, body mass index, kg/m2; WC, waist circumference, cm; VATA, visceral adipose tissue area, cm2; SATA, subcutaneous adipose tissue area, cm2; VATV, visceral adipose tissue volume, cm3; SATV, subcutaneous adipose tissue volume, cm3; LSR, liver-to-spleen attenuation ratio; PAFV, periaortic fat volume, cm3; EFV, epicardial fat volume, cm3; PaCFV, paracardial fat volume, cm3; SBP, systolic blood pressure, mmHg; FG, fast glucose, mmol/L; HbA1c, hemoglobin A1c, %; HDL-C, high-density lipoprotein cholesterol, mmol/L; LDL-C, low-density lipoprotein cholesterol, mmol/L.

Supplement Table 4: Body ectopic fat deposition in men and women with or without coronary artery atherosclerosis.

	Men 378			Women 372		
	**No/ CAD-mmCA (n=256)**	**CAD-msCA (n=122)**	**p-value**	**No/ CAD-mmCA (n=324)**	**CAD-msCA (n=48)**	**p-value**
Age, years	63.0 (62.1-63.9)	66.7 (65.4-68.0)	<0.001	64.7 (64.0-65.5)	69.0 (66.9-71.1)	<0.001
CAD, % (n)		34.1 (129)			12.6 (47)	
CAC score	16.1 (7.4-24.8)	558 (410-706)	<0.001	9.3 (5.4-13.2)	413.8 (204-623)	<0.001
Overweight, % (n)	46.8 (116)	48.1 (62)	0.416	37.5 (122)	51.1 (24)	0.044

Obesity, % (n)	13.3 (33)	19.4 (25)	0.063	11.1 (36)	10.6 (5)	0.520
Diabetes, % (n)	20.1 (50)	31.1 (40)	0.013	14.8 (48)	38.3 (18)	<0.001
Hypertension, % (n)	60.2 (150)	75.2 (97)	0.002	59.4 (193)	72.3 (34)	0.050
Current smoking, % (n)	48.6 (121)	37.2 (48)	0.022	2.8 (9)	2.1 (1)	0.633
Statin use, % (n)	10.0 (25)	20.9 (27)	0.003	9.8 (32)	21.3 (10)	0.025
HTN med, % (n)	39.0 (97)	61.2 (79)	<0.001	38.5 (125)	55.3 (26)	0.021
DM med, % (n)	10.0 (25)	19.4 (25)	0.010	4.9 (16)	19.1 (9)	0.002
SBP, mmHg	130.1 (128-131)	137.4 (134-140)	<0.001	132.6 (130-134)	140.0 (136-145)	0.004
BMI, kg/m^2	24.8 (24.4-25.2)	25.5 (24.9-26.1)	0.057	24.1 (23.7-24.4)	24.8 (23.9-25.6)	0.171
WC, cm	89.8 (88.6-91.0)	93.1 (91.5-94.8)	0.001	89.5 (88.4-90.7)	91.5 (89.1-93.8)	0.222
**Ectopic fat deposition**						
VATA, cm^2	136.3 (128-143)	156.3 (146-166)	0.002	110.9 (106-115)	132.5 (117-147)	0.002
SATA, cm^2	151.3 (143-159)	166.3 (154-178)	0.055	213.5 (203-223)	239.2 (220-257)	0.078
VATV, cm^3	2558 (2396-2720)	2884 (2656-3112)	0.021	1999 (1889-2109)	2585 (2517-3272)	<0.001
SATV, cm^3	1877 (1750-2004)	2137 (1938-2336)	0.054	2595 (2396-2795)	2854 (2477-3231)	0.279
LSR	1.24 (1.21-1.27)	1.21 (1.17-1.25)	0.282	1.28 (1.26-1.31)	1.26 (1.20-1.33)	0.671
Fatty liver, % (n)	23.7 (58)	24.2 (31)	0.501	18.9 (61)	24.4 (11)	0.246
PAFV, cm^3	21.3 (20.1-22.5)	24.7 (22.6-26.7)	0.004	12.4 (11.8-13.0)	15.9 (13.9-17.8)	<0.001
EFV, cm^3	56.4 (52.0-60.8)	65.1 (58.6-71.6)	0.027	35.8 (33.6-38.0)	44.0 (38.8-49.3)	0.010
PaCFV, cm^3	78.5 (74.3-82.8)	85.6 (79.1-92.1)	0.064	74.8 (71.2-78.4)	91.0 (80.9-101.1)	0.002
**Laboratory parameters**						
FG, mmol/L	5.70 (5.48-5.91)	6.44 (5.94-6.93)	0.002	5.59 (5.41-5.77)	6.85 (5.99-7.71)	<0.001
HbA1c, %	5.89 (5.78-6.00)	6.35 (6.12-6.57)	<0.001	5.90 (5.83-5.98)	6.38 (6.04-6.71)	<0.001
Triglyceride, mmol/L	2.14 (1.93-2.35)	2.03 (1.83-2.23)	0.495	1.75 (1.64-1.86)	1.98 (1.60-2.36)	0.152
HDL-C, mmol/L	1.24 (1.20-1.29)	1.16 (1.10-1.23)	0.035	1.53 (1.49-1.58)	1.42 (1.31-1.53)	0.087
LDL-C, mmol/L	2.97 (2.87-3.08)	3.13 (2.94-3.32)	0.111	3.27 (3.17-3.37)	3.17 (2.91-3.44)	0.503

Data are presented as mean (95% confidence interval, CI) for continuous and % (numbers, n) for categorical characteristics.

Abbreviations: CAD-CA, coronary artery atherosclerosis; CADmsCA, CAD moderate to severe coronary atherosclerosis; CADmmCA, CAD minimal to mild coronary atherosclerosis; BMI, body mass index, kg/m^2; WC, waist circumference, cm; VATA, visceral adipose tissue area, cm^2; SATA, subcutaneous adipose tissue area, cm^2; VATV, visceral adipose tissue volume, cm^3; SATV, subcutaneous adipose tissue volume, cm^3; LSR, liver-to-spleen attenuation ratio; PAFV, periaortic fat volume, cm^3; EFV, epicardial fat volume, cm^3; PaCFV, paracardial fat volume, cm^3; SBP, systolic blood pressure, mmHg; FG, fast glucose, mmol/L; HbA1c, hemoglobin A1c, %; HDL-C, high-density lipoprotein cholesterol, mmol/L; LDL-C, low-density lipoprotein cholesterol, mmol/L.

Supplement Figure 1. The representative sample images for body ectopic fat measurement.

1A

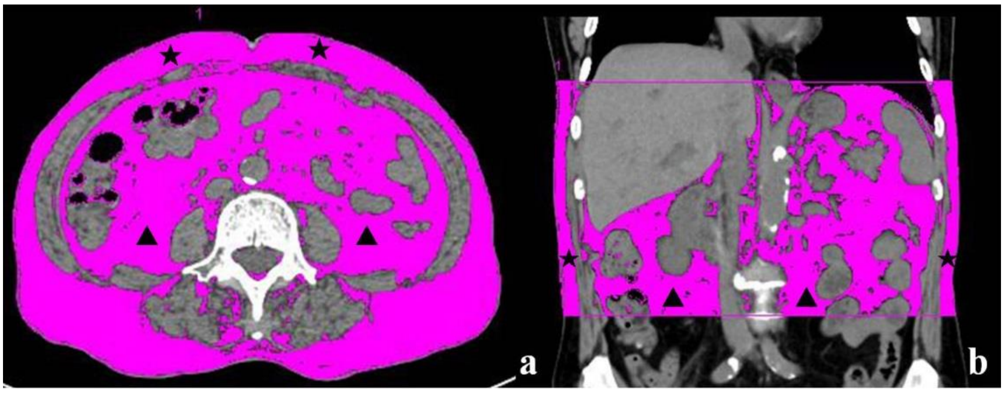

1B

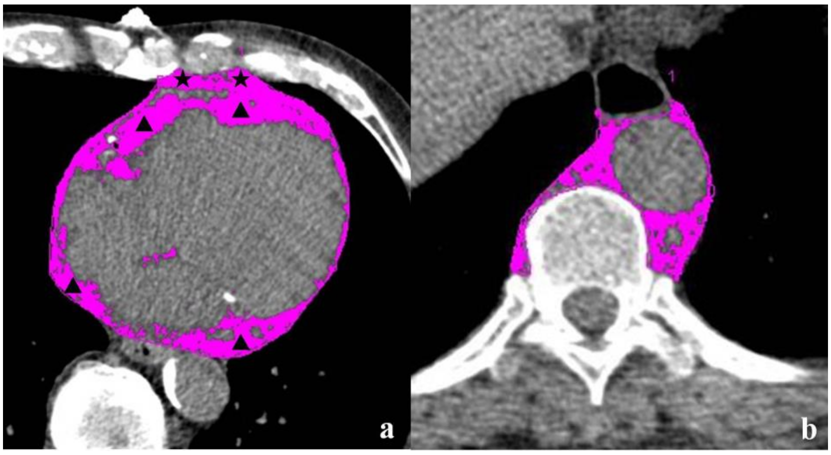

1C

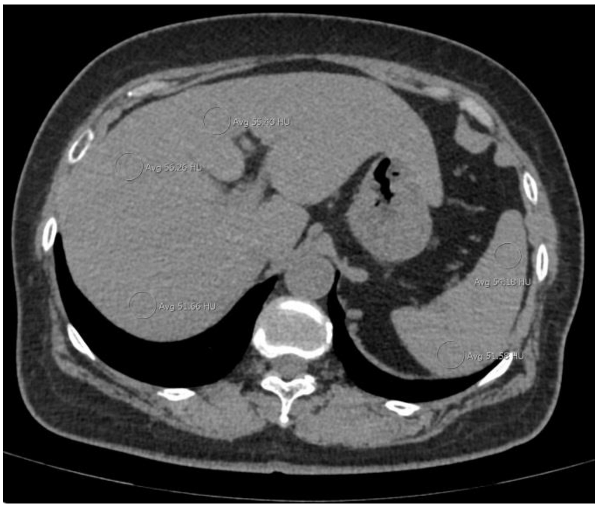

Supplement Figure 1A. Representative abdominal non-contrast-enhanced CT sample images for measurement a) visceral adipose tissue area (▲) and subcutaneous adipose tissue area (★); b) visceral adipose tissue volume (▲) and subcutaneous adipose tissue volume (★).

1B: Representative sample images for measurement a) epicardial fat volume (▲) and paracardial fat volume (★); b) periaortic fat volume.

1C. Representative sample images for measurement of fatty liver by liver and spleen attenuation in Hounsfield units.
